# FMRI-based identity classification accuracy in left temporal and frontal regions predicts speaker recognition performance

**DOI:** 10.1038/s41598-020-79922-7

**Published:** 2021-01-12

**Authors:** Virginia Aglieri, Bastien Cagna, Lionel Velly, Sylvain Takerkart, Pascal Belin

**Affiliations:** 1 Institut de Neurosciences de La Timone, UMR 7289, CNRS and Aix-Marseille Université, 13005 Marseille, France; 2 Department of Anesthesiology and Intensive Care, CHU Timone, Assistance Publique Hôpitaux de Marseille, Aix Marseille Université, 13005 Marseille, France; 3 Department of Psychology, Université de Montréal, Montreal, QC H2V 2S9 Canada

**Keywords:** Perception, Cortex

## Abstract

Speaker recognition is characterized by considerable inter-individual variability with poorly understood neural bases. This study was aimed at (1) clarifying the cerebral correlates of speaker recognition in humans, in particular the involvement of prefrontal areas, using multi voxel pattern analysis (MVPA) applied to fMRI data from a relatively large group of participants, and (2) at investigating the relationship across participants between fMRI-based classification and the group’s variable behavioural performance at the speaker recognition task. A cohort of subjects (N = 40, 28 females) selected to present a wide distribution of voice recognition abilities underwent an fMRI speaker identification task during which they were asked to recognize three previously learned speakers with finger button presses. The results showed that speaker identity could be significantly decoded based on fMRI patterns in voice-sensitive regions including bilateral temporal voice areas (TVAs) along the superior temporal sulcus/gyrus but also in bilateral parietal and left inferior frontal regions. Furthermore, fMRI-based classification accuracy showed a significant correlation with individual behavioural performance in left anterior STG/STS and left inferior frontal gyrus. These results highlight the role of both temporal and extra-temporal regions in performing a speaker identity recognition task with motor responses.

## Introduction

Our auditory system allows us to perform speaker recognition i.e. to recognize the identity of a familiar person simply based on her voice, presumably relying on speaker-voice associations formed in the past^1^ (as opposed to unfamiliar voice discrimination). Speaker recognition is, however, characterized by a considerable inter-individual variability^2^ with poorly understood neural bases. Several neuroimaging studies^3–10^ have sought to identify potential cerebral correlates of inter-individual variability in voice recognition ability: if a measure of cerebral activity can be predictive of a participant’s behavioural performance level that would be important both theoretically and for potential practical applications. Nakamura et al.^7^ using positron emission tomography during a speaker recognition task observed positive co-variations between task performance and cerebral blood flow in left frontal pole and left temporal pole. Andics et al.^8^ using fMRI during a speaker identification task observed that voice recognition performance co-varied with BOLD response in bilateral STS, right temporal pole and left amygdala. Schelinski et al.^9^ found, in healthy participants but not participants with autism spectrum disorder, that BOLD signal measured during a speaker recognition task in right STG correlated with behavioural performance at the task, but their analysis was restricted to temporal regions activated by the task.

The above studies examined correlations between regional indices of brain activity (mostly, fMRI -measured BOLD signal) and task performance. However, a more recent approach using multi-voxel pattern analysis (MVPA) compares the performance level of human participants at a given task with that of machine learning algorithms trained at the same task based on fMRI signal measured during the task. This approach is potentially more powerful as it probes the nature of information processing carried out in brain regions rather than their activity level. Bonte et al.^10^ applied this strategy to speaker recognition: they found that the accuracy of support vector machine (SVM) classifiers trained at the speaker categorization task, based on each participant’s fMRI signal measured in left superior temporal cortex (STC), explained a significant portion of the variance in behavioural speaker recognition level across participants. This pioneering result provides strong indication of the involvement of left STC in speaker recognition; however, the study was conducted in a relatively small group of 10 participants, encouraging confirmation in a larger sample; moreover the analysis was based on two large ROIs in temporal cortex, providing little neuroanatomical detail and leaving open the possibility that other areas, in particular in prefrontal cortex, may also predict behavioural scores. More recently, Ogg et al.^3^ found a significant correlation between instrument identification performance (but not speaker identification performance) and accuracy of SVM classifiers trained at speaker categorization in sound-sensitive regions.

Here we reasoned that the brain-behaviour relationship could potentially be better measured by sampling more equally the distribution of speaker identification scores in a larger group of participants, with comparable numbers of subjects in the extreme and middle parts of the distribution, rather than relying on a random selection procedure that would necessarily inflate the proportion of subjects with average performance compared to those with very high or very low scores. Hence, the aim of the current study was to analyse fMRI data collected during a speaker recognition task using MVPA methods (searchlight analysis^11^) in order to investigate the distributed representational pattern of speaker identification processes, in a larger sample of subjects (N = 40) selected for their wide distribution of voice recognition abilities.

We first asked whether some sound-responsive cortical regions would yield above chance classification of the speaker identity by fMRI-based classifiers. We expected to replicate previous results in observing the involvement of temporal areas, but wanted to further investigate the implication of prefrontal areas, as the only study so far having observed a correlation between fMRI-based classification and participant performance restricted its analysis to auditory cortical areas of the temporal lobe^10^. Furthermore, in order to check that the regions in which speaker classification was higher than chance were actually classifying speakers’ voice independently of the actual word, a ‘generalization’ analysis was performed in which training and testing of the classifiers was performed on different words. We then asked whether fMRI-based classification accuracy within sound-responsive cortical regions could predict individual differences in voice recognition abilities.

## Methods

### Subjects

The first step for subjects selection was to send an online version of the Glasgow Voice Memory Test^2^ through social media and experimental psychology mailing lists. This test was periodically advertised until a performance distribution similar to the one previously observed^2^ was obtained. Then, all 209 subjects that took the GVMT online were contacted to come to the laboratory for a remunerated experimental session in which they had to complete a speaker identification task (the term identification is here preferred since the task consists in a one-to-one association between a speaker and a finger press). Exclusion criteria included having an auditory impairment. Among these 209 subjects, 86 came to the laboratory for the first behavioural session (54 females; mean age ± SD = 24.9 ± 5.7). Prior to testing sessions, participants provided written informed consent, following the guidelines of the declaration of Helsinki and with the approval of the Comité de Protection des Personnes Sud Méditerranée I (ref. 16 78).

In order to try to select subjects with a flat distribution of voice recognition abilities, optimal for investigating the neural correlates of inter-individual variability in behavioural identification performance, the scores obtained at the identification task by the 86 subjects during the laboratory session were divided in four arbitrarily defined performance bins (see Table 1 for details on subjects’ selection). Then, we endeavoured to first recruit subjects belonging to the two extreme classes for the MRI session; then, the subjects belonging to the average classes were contacted. Eventually, 40 subjects (28 females; M ± SD = 25.3 ± 5.5, range = [18–41]) were successfully recruited for the MRI session. A French translation of the Edinburgh Handedness Inventory was administered before the MRI session (M ± SD = 0.78 ± 0.27, range = [− 0.1 1]; a score of 1 indicates strong right-handedness, while -1 strong left-handedness). The South Mediterranean Committee for experimental ethics approved the methodology and the experimental protocol used in the study.

**Table 1:** Selection of subjects for fMRI session.

Score range—laboratory session	[10 32]	[32 54]	[54 76]	[76 98]
Number of subjects in the percentile	1	14	40	31
Number of subjects selected for fMRI	1	6	16	17

1st row: upper and lower boundaries of each class of performance obtained in the laboratory testing session (no feedback session); 2nd row: number of subjects in the corresponding class; 3rd row: number of subjects selected for the fMRI session.

### Stimuli

Three French-Canadian female voices were selected among a high-quality database of vocal recordings^12^. Original recordings were made in the multi-channel recording studio of Secteur ElectroAcoustique in the Music faculty of Université de Montréal. The speakers were instructed to perform multiple vocalizations (e.g. stories, words, non-verbal vocalizations, sing…). For this experiment, two short stories and 12 disyllabic French words (“français”, “allô”, “jeudi”, “lundi”, “mardi”, “neutre”, “partie”, “penser”, “pouvez”, “quatre”, “toussez”, “voyelle”) were selected by manually cutting them in Adobe Audition (© 2016 Adobe Systems Incorporated); residual background noise was then attenuated through the automatic healing procedure offered in Adobe Audition and energy of the stimuli was normalized through root mean square process using custom code in Matlab R2014B (The MathWorks, Inc., Natick, MA, USA). Table 2 shows a summary of basic acoustical parameters for the stimuli. Fundamental frequency (F0), formant dispersion (average difference between first four formants) and harmonics-to-noise ratio (HNR) have been extracted in Praat^13^ for three sustained vowels [a, i, u]. F0 variability (standard deviation of F0) has been also extracted for each word through the STRAIGHT algorithm^14^ in order to obtain a measure of speakers’ F0 variability across the different words.

**Table 2:** Acoustical parameters of the stimuli presented in the task.

	Anne (42 YO)		Betty (19 YO)		Chloe (56 YO)	
	Mean	SD	Mean	SD	Mean	SD
Words duration (sec)	0.59	0.12	0.50	12.12	0.65	0.15
F0 (vowels)	175.68	5.60	230.25	19.92	254.39	48.07
F0 variability (words)	37.93	16.69	24.64	7.94	63.02	35.46
HNR (vowels)	15.19	5.84	19.10	1.56	18.57	4.86
FD (vowels)	1134.67	286	1095	95.99	1143.26	137.40

*F0* fundamental frequency, *HNR* harmonics to noise ratio, *FD* formant dispersion.

### Experimental design: laboratory session

Subjects performed a speaker identification paradigm (inspired by the study of Bonte et al.^10^) built in Psychtoolbox^15–17^ and running on Matlab R2014B. After ensuring that subjects understood the task, they were seated 60 cm from the screen (BENQ 2.52 × 21.92 × 14.28 inches). The sounds were delivered through headphones (Beyerdynamics DT770 pro, beyerdynamic GmbH & Co. KG, Heilbronn, Germany).

#### Learning phase

In this phase subjects had to familiarize with three speakers (see Supplementary Information for details on speakers’ voices) by initially listening to two short stories per speaker (mean duration (sec) ± SD = 11.0 ± 0.8), while memorizing the association between the speaker’s voice and name printed on the screen. Speakers’ names were chosen arbitrarily in such a way to have no more than two syllables and start by the 3 first letters of the alphabet (Anne, Betty and Chloe). Subjects were instructed not to pay attention to stories’ content (identical across speakers) but rather to try to memorize the speaker’s voice and the association with her name. After story presentation, twelve disyllabic words recorded for each of the 3 speakers were delivered in a pseudo-randomized order created through Mix and Match^18^, with the only constraint being not to exceed two repetitions in a row of the same speaker. After listening to a voice, subjects had to identify the correct speaker by pressing one of the three keys on a keyboard built appositely for the experiment (thumb = Anne; index = Betty; middle finger = Chloe). Immediately after subject’s button press, a feedback message was provided for both correct recognition (“Yes!” in green characters) and misidentification (“No! It was -*name of correct speaker*- in red characters); after a wrong answer, the misidentified sound was played while the feedback message was on the screen. Afterwards, the subject pressed a button to listen to the following voice. A 2-s interval separated key press and the following stimulus. No time restriction was applied on subject’s answer. Both stories presentation and learning phase were repeated twice, the two stories being delivered in the same order during the two sessions and words in a different pseudo-randomized order. Hence, during this phase, a total of 72 identification trials were presented (12 words * 3 speakers * 2 sessions).

#### Testing phase

Upon completion of the training session, subjects were asked to repeat the same task learned in the first two sessions but without receiving a feedback on their answer. Again, response time was self-paced. At the end of the testing session, the mean percentage of correct responses obtained in the two training sessions and in the testing session appeared on the screen.

### Experimental design: fMRI session

#### Training outside scanner

On the day of the fMRI session participants had to first complete a training session to further familiarize with the identification task. First, they were presented with one learning session as in the laboratory. Then, they had to perform two testing sessions (no feedback provided) while an audio recording of the EPI scanning noise was added at diminished amplitude (− 5 dB) as a background noise on the stimuli. Furthermore, subjects were instructed to keep their eyes closed while performing the two testing phases and to answer within 5 s following sound presentation to keep training session as similar as possible to MRI session.

#### fMRI acquisition

All the acquisitions were carried out in a 3 T Prisma MRI scanner (Siemens, Eerlangen, Germany) with a 64-channels head coil at the fMRI centre of the Institute of Neuroscience of La Timone in Marseille, France. Both functional and structural images were acquired following guidelines of the Human Connectome Project acquisition protocol^19,20^. Scanning session started with the acquisition of a phase-reversed pair of spin echo images with the same geometry as fMRI data (Field of View = 240 × 240 mm^2^, slice thickness = 2.5 mm, TR = 677 ms, TE = 4.62 ms, encoding phase = anterior to posterior) that were used to calculate a field map for correction of B0 inhomogeneities.

During functional scanning, subjects completed the same identification task as during training (without the feedback condition) while they were lying down in the scanner equipped with Sensimetrics S14 MR-compatible insert earphones, connected to a Yamaha P-2075 power amplifier. Auditory stimuli were delivered at 85–95 dB SPL using a software developed for this study in the National Instruments LabVIEW 2014 environment; this software was driven by the MR pulses using a NI-PXI 6289 digital input/output hardware, which also allowed recording subjects’ responses, obtained by means of a digital ergonomic 5-keys keyboard.

**Figure 1:**
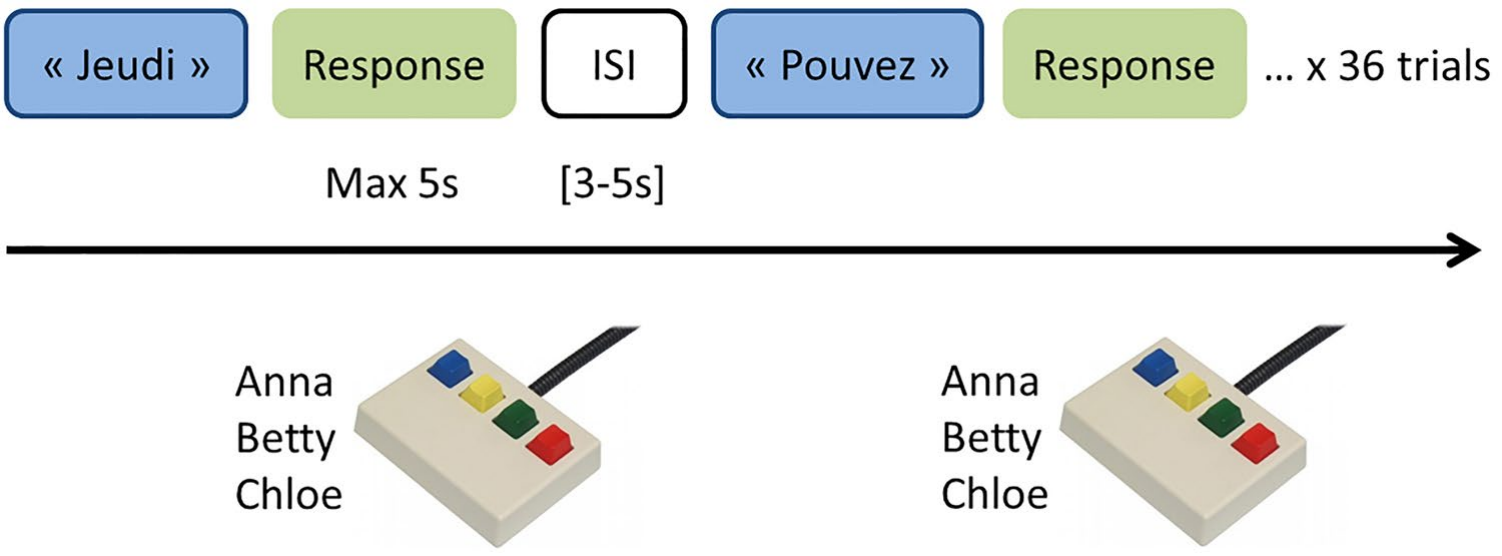
Identification task (fMRI version). In each trial, a French word was delivered (e.g. “Jeudi”) and subjects had to recognize the speaker by pressing one of three keys on a five-keys keyboard (“Response”) within a 5-s window; the following trial (e.g. “Pouvez”) was then delivered after an interstimulus interval (ISI) randomized between 3 and 5 s. One run was made up of 36 trials.

Functional scanning for the identification task consisted of four functional runs, each including 36 trials (12 disyllabic words * 3 speakers) in which participants had to identify the speaker by button press, as previously learned. Subjects were instructed to listen to the words with eyes closed and to recognize the speaker by pressing the corresponding button within the 5 s following voice delivery. In case of missing response during this time window, the following stimulus was delivered. The inter-stimulus interval (ISI), measured as the interval between the response instant (or the end of the 5-s answer window in case of missing response) and the delivery of the following stimulus, varied between 3 and 5 s (see Fig. 1); ISIs were generated according to an exponential distribution and randomized across the runs^21^. Each run lasted 5 min and 62 s, resulting in total task duration of approximately 25 min.

After the identification task, participants also underwent one single run of an event-related voice localizer paradigm in which they were asked to close their eyes while simply listening to vocal and non-vocal sounds. 144 sound stimuli of a fixed duration of 500 ms were delivered. There were six categories of sounds, each containing 24 stimuli: vocal/verbal, vocal/non-verbal, vocal/emotional, non-vocal/animal, non-vocal/environmental and non-vocal/artificial. Most of the stimuli (95%) belonged to a database created for a previous study^22^, while others were downloaded from public databases free of copyright. A sequence of randomized ISIs of a duration that varied between 4 and 5 s separated each stimulus.

The order of the stimuli in both fMRI tasks was pseudo-randomized by an optimization process which allows maximization of BOLD response^23,24^; four different orders, one for each run, were created for the identification task and kept constant across subjects while one single order was used for the localizer task.

Functional scans were acquired with a multi-band gradient echo-planar imaging (EPI) sequence with whole-brain and cerebellar coverage (TR = 955 ms; multiband factor = 5; voxel size = 2 × 2 × 2 mm^3^; 60 slices).

The TE was of 35.2 ms, with a flip angle of 56 degrees and a field of view (FOV) of 200 × 200 mm^2^; matrix of acquisition was 100 × 100. After EPI images acquisition, a high resolution 3D T1 image was acquired for each subject (voxel size = 0.8 × 0.8 × 0.8 mm^3^, TR = 2400 ms, TE = 2.28 ms, FOV = 256 × 256 mm^2^). In total, 366 EPI volumes were acquired in each run of the identification task and 792 in the voice localizer task. Furthermore, a single-band reference image (SBREF image) was acquired without slice acceleration during the few seconds of reference scans before first stimulus delivery so that it could be used for motion correction and realignment of functional and structural images. Total acquisition time (field map, EPI sequences, T1) was of ~ 45 min.

At the end of the fMRI session, subjects were requested to answer different questions regarding the comfort in the scanner as well as their impressions on the identification task (e.g. difficulty, influence of the scanner noise, strategy to recognize speakers). Furthermore, they had to list all the words pronounced by the three speakers so that this measure could be correlated to the performance obtained at speaker identification.

The raw data, design for fMRI experiment (timing, events) and subjects’ responses (choice and RT) in the laboratory and during MRI session are available on-line (https://openneuro.org/datasets/ds001771/versions/1.0.2; https://zenodo.org/record/2591038#.XrA0pZ4zapo)^25^.

### Preprocessing of fMRI data

All preprocessing steps and model specification were performed in SPM12b MRI (r6080—Wellcome Department of Cognitive Neurology, University College London). The phase difference between acquisition encoding directions and the magnitude field map were used to compute a Voxel Displacement Map (VDM) to correct for distortions of EPI images. At this point, the realigned SBREF image of the first run was coregistered to subject’s T1 (which was previously coregistered to an MNI template). BOLD images of the four runs were then realigned to the first scan, resliced and corrected for distortions using the VDM. Afterwards, BOLD images of all runs were coregistered to the realigned and MNI-coregistered SBREF image of the first run. In order to obtain a subject-specific template, subjects’ T1 images were segmented into the native tissues (white and grey matter), which were in turn iteratively coregistered with all other subjects to obtain the DARTEL template. These two steps were performed through the segmentation algorithm included in SPM12, which uses diffeomorphic registration to preserve cortical topology applying a membrane bending energy or Laplacian model^26^. Normalization parameters (flow fields) were also created at this step and then used to normalize the realigned and unwarped BOLD images of the localizer task (on which univariate analysis were performed) to the DARTEL template and finally to the MNI space (affine registration). In the normalization step, carried out only for the images undergoing univariate analyses, a Gaussian smoothing kernel of 1 mm has been applied to avoid aliasing. A further Gaussian smoothing kernel of 4 mm was then applied to these normalized images. One subject was excluded from further analysis because more than 20% of the functional volumes in both tasks were characterized by rapid scan-to-scan motion based on changes in translation and rotation parameters higher than 0.5 mm and 3 standard deviations from the mean, resulting in a total sample of 39 subjects.

### Univariate analysis

In order to check that the contrasts voices *vs* non-voices and voices *vs* baseline were consistent with results obtained in previous fMRI studies investigating voice perception, as well as to obtain an explicit mask of sound-responsive regions specific to our sample, classical univariate analyses were carried out for both the voice localizer and the identification task. Normalised and smoothed EPI images were inserted in a subject-specific design matrix defining the first-level general linear model (GLM). For the speaker identification task, the matrix contained one regressor per speaker modelling the duration of vocal stimulation. An additional regressor, modelled with duration of 0 s, accounted for subject’s button pressing. Voice localizer GLM contained 6 regressors (one per sound category). In both GLMs, six realignment parameters and twenty-six noise regressors resulting from the denoising procedure carried out with the Physio Toolbox^27^ were also added. These twenty-six noise regressors included the components resulting from a Principal Component Analysis (PCA) on the segmentation of “noise ROIs” (12 for white matter and 12 for CSF) and the corresponding two average values. Finally, there was one constant term per run (one in the voice localizer, four in the identification task). Voices, button press for the identification task as well as sound regressors for the voice localizer task were obtained by convolving boxcar functions representing the onsets and durations of stimulation blocks with the canonical hemodynamic response function (HRF). Before model estimation, each voxel’s time course was filtered through a high-pass filter at 128 s and auto-correlation was modelled using an auto-regressive matrix of order 1. After model estimation, the contrast between the three speakers’ voices and baseline was computed for the identification task and the contrasts between sound and baseline as well as between vocal or non-vocal stimuli and baseline for the voice localizer. To look at group results, the images of the first level contrasts of all 39 subjects were inserted in one-sample t-tests to assess where BOLD signal change was significantly different from zero (*p* < 0.05, corrected for family-wise error (FWE) at the voxel level and *p* < 0.001 uncorrected for multiple comparisons to build a region of interest for MVPA analysis from voice localizer). Where of interest, significant clusters were then anatomically labelled according to the SPM Anatomy toolbox^28^.

### MVPA searchlight analysis

The GLM for the multivariate analysis of the identification task included 36 single regressors (one per each word heard in a run) and one regressor for response (duration = 0 s). As for the univariate GLMs, in the multivariate model there were also six realignment parameters, twenty-six noise regressors and one constant term per run. The model was estimated on non-normalized and unsmoothed images.

MVPA searchlight analysis was performed in native space images using Nilearn, a Python package that uses scikit-learn library^29^. A whole brain searchlight was performed on single trial beta maps for each subject to map the classification accuracy of speaker identification by centring a spherical neighbourhood with a 12 mm radius (6 voxels radius, about 900 voxels per sphere) (similar to the choice of 10 mm in Correia et al.^30^) at each voxel of the subject-specific whole-brain mask and employing a linear Support Vector Machine (SVM—radial basis function kernel with soft margin parameter C = 1) on betas from all voxels contained in the sphere.

Since there were three classes that needed to be classified (multiclass problem), we performed a one-vs-one (OvO) reduction, in which the classifier learns to classify pairs of speakers in the training set (e.g. Anne *vs* Betty, Betty *vs* Chloe, Anne *vs* Chloe); then, when applying the classifier in the data set, the class receiving the highest number of predictions is assigned to the voxel. Since there were four runs, the cross-validation was of the type “leave-one-run out”, consisting in training the classifier on three runs and testing it on the remaining one.

This first step yielded one map representing the classification accuracy score per subject. Chance level (33%) was then subtracted from these maps, which were then normalized to the Dartel template and smoothed (Gaussian smoothing kernel of 8 mm) before being inserted in a non-parametric permutation-based group analysis (with N permutation = 5000) (SnPM13; https://warwick.ac.uk/fac/sci/statistics/staff/academic-research/nichols/software/snpm^31^) assessing in which regions classification accuracy was significantly higher than chance and correlated with behavioural performance. For a given voxel in MNI space, a distribution of accuracy values was generated by randomly adding or subtracting the value of each individual participant at each iteration (n = 5000, a common guideline is that 1000 permutations are sufficient to properly characterize the permutation distribution). The correct accuracy value obtained by adding all participant values is then compared to the distribution. Since the aim of the study was to understand in which sound-responsive regions speaker classification took place and in which of these regions there was a significant correlation with behavioural scores, an explicit mask of the regions that were significantly activated during auditory stimulation (at a liberal threshold of *p* < 0.001, uncorrected for multiple comparisons; Fig. 3) during the voice localizer task was applied to all multivariate group results. This analysis resulted in a group-level map of sound-responsive voxels showing significantly (p-FWE < 0.05 voxel-level-corrected) above-chance fMRI-based identity classification accuracy.

We also performed a complementary, ‘generalization’ analysis using a ‘leave-one-word-out’ classification scheme. SVM classifiers were trained on data for 11 words from each speaker (33 stimuli × 4 runs) then tested on data for the remaining word (1 stimulus × 4 runs)—1 different word for each fold of a 12-fold cross-validation scheme. The searchlight analysis yielded no voxel with significantly (FWE-*p* < 0.05) above-chance accuracy at the group-level. We then performed a simpler region of interest (ROI) analysis in which training and testing was performed using fMRI data from all significant voxels of the group-level accuracy map instead of local spheres, yielding one accuracy value per participant. Comparison of accuracies between standard and leave-one-word-out classification schemes was performed using a paired t-test; comparison with chance-level using a one-sample t-test.

## Results

### Behaviour

Behavioural results obtained in the laboratory and training sessions are reported in the Supplementary Information file.

**Figure 2:**
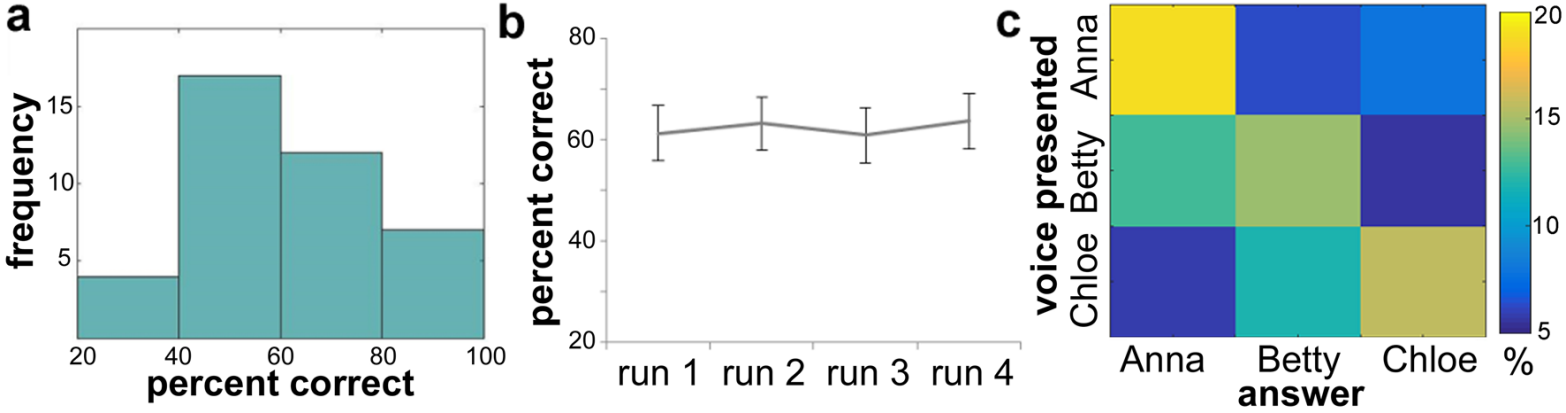
Behavioural performance during the fMRI session. a. distribution of the scores obtained in the scanner (N = 40); b. mean performance across the four runs. Error bars represent 95% confidence interval. c. Confusion matrix of presented voices vs. answers. Colorscale indicates proportion of trials corresponding to each Presented voice-Answer pair. The sum of the 9 cells is 100%; random performance would be 11.11% in each cell; perfect performance would be 33.33% in each cell of the diagonal and zero elsewhere.

Identification scores obtained in the fMRI session (N = 40) are reported in Table 3 and their distribution is shown in Fig. 2. There was a main effect of speaker on both PC scores (*F* (2, 78) = 6.01, *p* = 0.003, η^2^ = 0.13) and reaction times (*F* (1.61, 62.81) = 6.23, *p* = 0.006, η^2^ = 0.14) while percentage of missing responses for different speakers were not significantly different (*F* (2,78) = 1.05, *p* = 0.35, η^2^ = 0.03) There was no main effect of run on PC scores (*F* (3, 117) = 1.60, *p* = 0.19, η^2^ = 0.04), nor on reaction times (*F* (2.60, 101.44) = 1. 20, *p* = 0.29, η^2^ = 0.03), nor on percentage of missing responses (*F* (3, 117) = 1.22, *p* = 0.30, η^2^ = 0.03), meaning that subjects were stable in their performance across the different runs. The interaction between run and speaker effects was not significant: the recognition rate specific for each speaker remained constant across runs (*F* (5.48, 213.73) = 1.14, *p* = 0.34, η^2^ = 0.03), as well as the reaction times (*F* (6, 234) = 1.33, *p* = 0.24, η^2^ = 0.03) and the percentage of missing responses (*F* (6, 234) = 0.91, *p* = 0.50, η^2^ = 0.02). A post-hoc Tukey test revealed no significant differences between PC for Anne’s and Betty’s voices (*p* = 0.81), while Chloe’s voice was recognized significantly less accurately than both Anne’s (*p* = 0.004) and Betty’s voice (*p* = 0.02). As for reaction times, participants were significantly slower in recognizing Betty as compared to Anne (*p* = 0.006), while the other comparisons were not significant. Figure 2c shows the 3 speakers × 3 answers confusion matrix averaged across all runs and subjects. The diagonals of the matrix correspond to the values of Table 3, while off-diagonal cells indicate the pattern of errors: most errors consisted of confusion between Betty and Chloe.

**Table 3:** Behavioural results.

	PC identification	Reaction time (s)
Anne	66.3 ± 20.0	1.2 ± 0.5
Betty	63.7 ± 16.6	1.3 ± 0.5
Chloe	56.5 ± 16.3	1.3 ± 0.4
Total	62.4 ± 16.1	1.3 ± 0.5

Mean percent correct scores (PC) with relative standard deviation averaged and mean reaction times with relative standard deviations obtained in the fMRI session (averaged over the four runs).

### Univariate analysis

**Figure 3:**
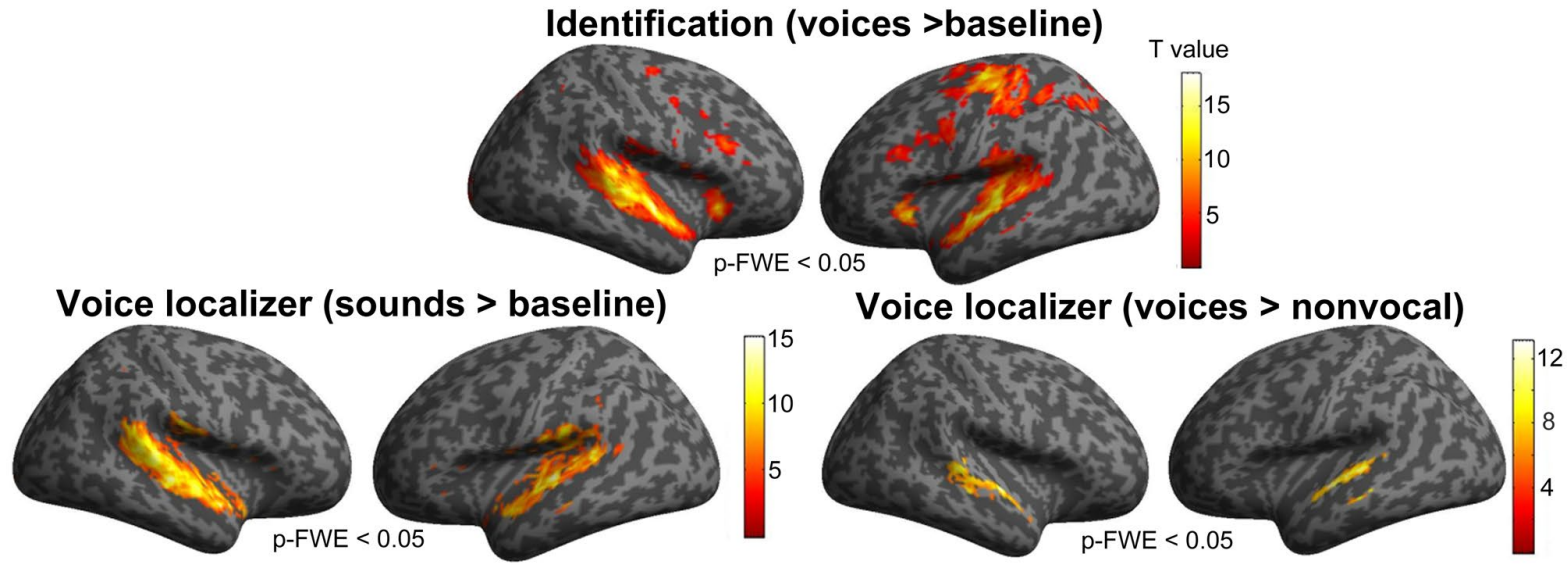
Univariate random effects (RFX) analysis. First row: identification task, contrast voices > baseline; second row: localizer task, contrast sound > baseline (left) and voices > non-voices (right). All contrasts are shown at *p* < 0.05 FWE-corrected (voxel-level), extent threshold = 0 mm^3^. The group statistical map is here overlaid on an inflated render in SPM.

The results of the univariate contrast voices > baseline obtained for the identification task show distributed activations in auditory regions as well as TVAs along the STS/STG but also in bilateral inferior frontal gyrus (IFG), bilateral precuneus, motor and premotor regions and bilateral cerebellum (Fig. 3 and Table 4). As for the voice localizer, the contrast all sounds > baseline also resulted in the activation of bilateral auditory regions as well as prefrontal regions, while the contrast voices > non-voices highlighted bilateral TVAs along STS/STG (Fig. 3).

**Table 4:** Random effects analysis for the identification task (contrast all voices > baseline) at *p* < 0.05 FWE-corrected, extent threshold = 100 mm^3^.

Coordinates (x y z)	t	Cluster size	Anatomical label
58 − 4 − 6	18.04	4197	Right STG
− 58 − 6 − 2	17.08	3820	Left STG
− 30 22 2	16.64	412	Left Insula
12 − 30 − 4	16.44	5091	Parahippocampal gyrus
− 4 12 50	15.88	1981	Left middle frontal gyrus
− 36 − 18 62	14.44	3658	Left precentral gyrus (PrcG)
30 24 0	13.41	407	Insular cortex
− 28 − 60 − 24	12.17	431	Left cerebellum*
− 6 − 90 0	11.81	2109	Left calcarine gyrus
42 18 24	10.23	259	Right IFG (triangularis)
− 40 6 28	9.89	564	Left IFG (opercularis)
38 − 12 62	8.95	238	Right PrcG

The 1st column of the table reports the MNI coordinates of local maxima (peaks separated by more than 8 mm); the 2nd column contains t-values estimating the contrast of interest (height threshold t (1, 38) = 6.15, *p* < 0.05 FWE corrected); the 3rd column reports relative cluster size in voxels; the 4th column contains the anatomical locations of the corresponding cluster. *Cerebellum is not shown in the render.

### MVPA searchlight analysis

**Figure 4:**
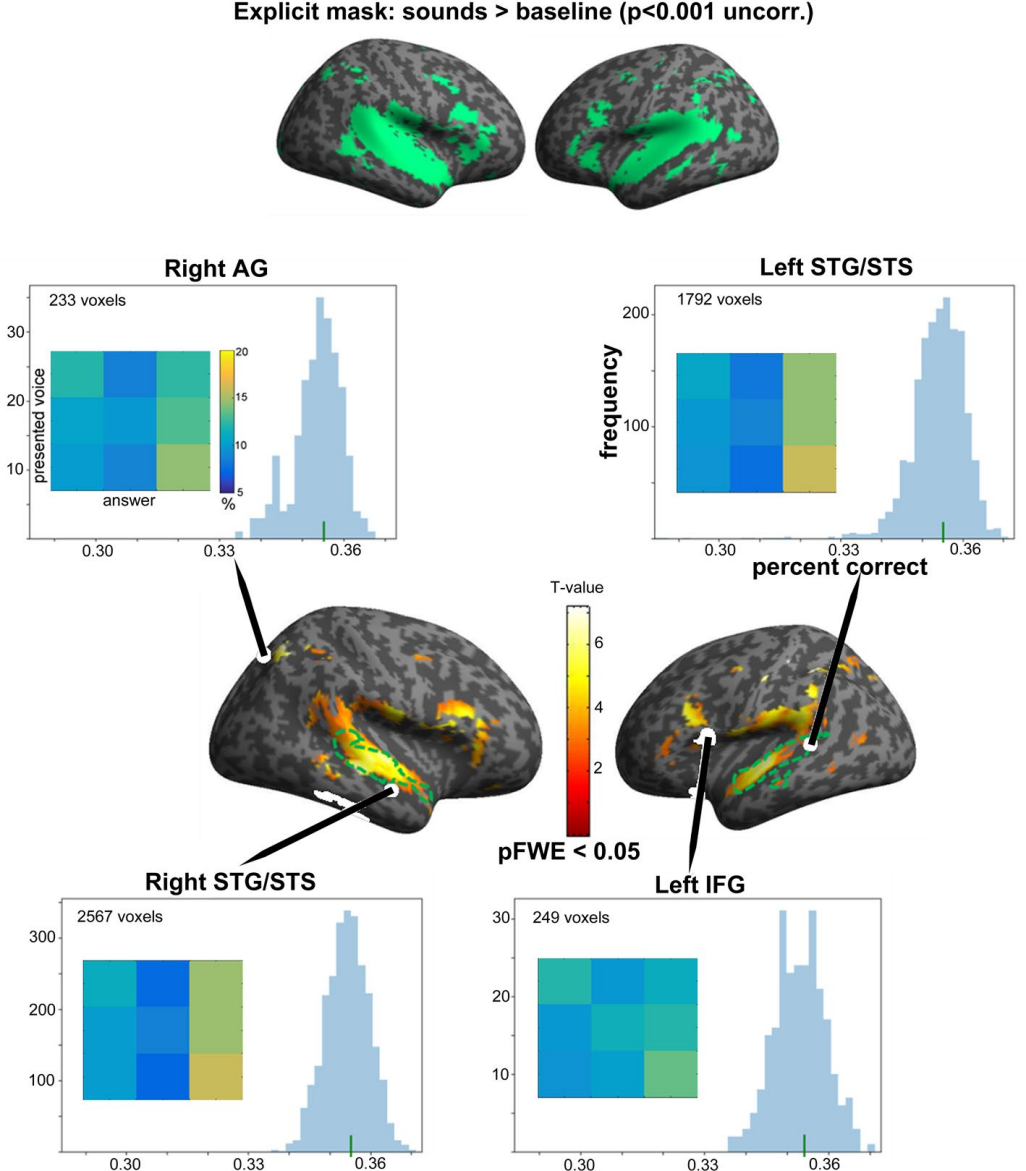
Maps of significant group-averaged above-chance speaker classificationaccuracy in sound sensitive regions. Upper row: in green, region of interest (ROI) used as explicit mask (voxels showing significant higher activity for sounds as compared to baseline in the voice localizer task at *p* < 0.001 uncorrected for multiple comparisons). Below: results of the group-level permutation-based random effects analysis showing the regions in which classification accuracy was significantly higher than chance (p-FWE < 0.05 voxel-level-corrected) in the region of interest. Green dashed contours indicate TVAs obtained in the sample (V > NV). The group statistical map is here overlaid on an inflated render in SPM and shown at uncorrected level (*p* < 0.00002) for illustration purposes. The histograms show the distribution of speaker classification accuracy scores across all voxels, for the clusters with more than 200 voxels. Green vertical lines represent median classification accuracy scores across voxels. Matrices represent averaged confusion matrices yielded by the classifiers at each cerebral location, with same convention as the behavioural confusion matrix of Fig. 2c.

Speaker classification accuracy by the SVM classifier was significantly higher than theoretical chance level (33%) for 12-mm spheres centred in voxels of multiple cortical areas, including left and right STG/STS, left supramarginal gyrus (SMG), right angular gyrus (AG), left IFG (triangularis) and left pre/post-central gyrus (Fig. 4 and Table 5). Variability of fMRI-based classification accuracy across voxels within selected clusters is shown in the histograms in Fig. 4. Figure 4 also includes confusion matrices yielded by the classifiers at each of the four locations. None of these fMRI-based confusion matrices correlated with the subjects’ average behavioural confusion matrix, although left IFG came close (rho = 0.52, *p* = 0.081 one-tailed; other rhos < 0.15 *p* > 0.35).

**Table 5:** Non-parametric random-effects analysis assessing fMRI-based SVM speaker classification accuracy at *p* < 0.05 FWE-corrected, extent threshold = 20 mm^3^.

Coordinates (x y z)	t	Cluster size	Anatomical label
− 46 − 36 48	7.67	94	Left inferior parietal lobule (IPL)/supramarginal gyrus (SMG)
34 − 56 52	7.30	233	Right superior parietal lobule/angular gyrus
− 48 − 14 50	7.27	92	Left postcentral gyrus
− 58 − 12 4	7.23	1809	Left STG
58 − 10 − 2	6.99	2591	Right STG
− 42 20 24	6.42	249	Left IFG (triangularis)

The 1st column of the table reports the MNI coordinates of local maxima (peaks separated by more than 8 mm); the 2nd column contains t-values assessing that classification accuracy scores across subjects was higher than 33% (height threshold t (1, 36) = 4, *p* < 0.05 FWE voxel-size corrected); the 3rd column reports relative cluster size; the 4th column contains the anatomical location of the clusters.

We next asked whether these areas would also yield above-chance classification accuracy in a more abstract classification scheme in which training and testing is performed on different words—the ‘generalization’ test^32^. In doing so, an SVM classifier was trained and tested in a ‘leave-one-word-out’ classification scheme, which is, tested on a different word based on BOLD signal from all voxels having yielded significantly (*p* < 0.05 FWE-corrected) above-chance accuracy at the group-level. Classification accuracy significantly dropped compared to the ‘leave-one-run-out’ scheme in this more conservative test of recognition (*t* = 2.64, *p* = 0.01) but remained significantly above chance (Mean classification accuracy ± SD = 34.92 ± 4.34, *p* < 0.02).

### Correlation between classifier’s and participants’ accuracy

**Figure 5:**
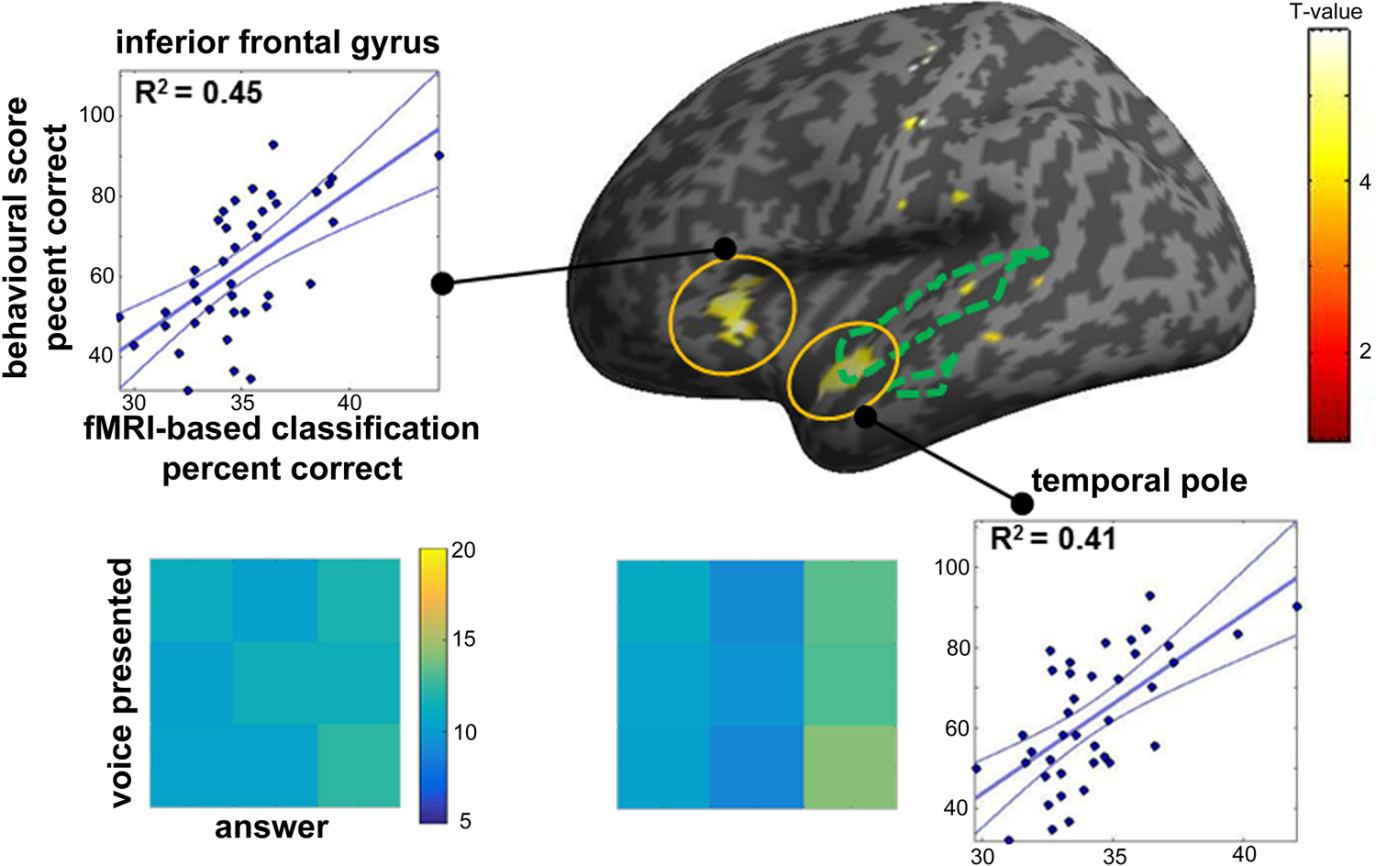
Correlation of speaker classification accuracy with individual speaker recognition scores. Co-variation of speaker classification accuracy with identification scores in voice sensitive regions was computed in a permutation-based regression model with one covariate. R^2^ = coefficient of determination of Spearman correlation coefficient. Confusion matrices are sown with the same convention as in Fig. 2c. Green dashed lines indicate TVAs obtained in the sample (V > NV). The group statistical map at an uncorrected threshold (*p* < 0.001) is here overlaid on an inflated render in SPM for illustration purposes.

Next we asked whether there would be sound-responsive regions in which fMRI-based classification accuracy would correlate with individual participants’ speaker identification score during scanning, by means of a regression model with one covariate. This analysis performed across the 4 runs resulted in significant correlations (*p*-FWE corrected at voxel-level < 0.05, extent threshold = 10 mm^3^; Fig. 5) in left IFG (x, y, z: -40, 22, 8; *t* (1, 37) = 5.08; cluster size = 23) and left temporal pole (x, y, z: -50, 14, -18; *t* (1, 37) = 4.94; cluster size = 21), including voxels belonging to the group-specific TVAs (see green dashed contour in Fig. 5). However confusion matrices derived from fMRI-based categorisation correlated only weakly with the behavioural confusion matrix (Left temporal pole: rho = 0.167; one-tailed p-value = 0.326 n.s.; Left Inferior prefrontal gyrus: rho = 0.233 *p* = 0.266 n.s.).

## Discussion

This study was aimed at clarifying the cerebral correlates of familiar speaker recognition (as opposed to unfamiliar speaker discrimination) as well as the link between behavioural and neural individual differences in voice perception processes. MVPA methods were applied to fMRI data acquired during a speaker identification task in a sample of 39 subjects previously selected on the basis of their widely distributed performance level at that same task. The results showed that a network of regions including bilateral TVAs and parietal regions (SMG) and left frontal and prefrontal regions yielded significantly above-chance speaker classification accuracy scores as measured through a searchlight MVPA analysis. Interestingly, subjects showing higher fMRI-based classification accuracy within left anterior STG and IFG also obtained higher scores at the behavioural speaker identification task, as reflected by a significantly positive correlation (R^2^ = [0.41 0.45]).

### An extended network for processing speaker identity

FMRI-based speaker classification accuracy was significantly higher than chance in clusters along bilateral STG/STS and parietal regions, and left prefrontal cortex. “Voice patches” are regions along the STS/STG (anterior to posterior) well known to be involved in processing vocal stimuli, both during voice perception and speaker identification tasks^10,32–35^. Here, we demonstrated that the “voice patches” specific to our analysis do not only show preferential activity for vocal than non-vocal sounds but they also contain information allowing significantly above-chance speaker identity classification by the SVM. The largest cluster showing significant speaker classification accuracy was the anterior/middle portion of the right STG/STS, a region that consistently showed sensitivity to speaker identity^36–39^.

Interestingly, regions leading to above-chance fMRI-based speaker classification were also observed in left frontal cortex (IFG), left post-central gyrus and bilateral parietal regions (SMG/AG). To our knowledge, this is the first study reporting the involvement of extra-temporal regions in a speaker classification task involving overt motor responses, as revealed by MVPA. Bonte et al.^10^ previously employed MVPA to unravel the regions classifying speaker identity and speech content, but their analysis was limited to the superior temporal cortex. Here, the explicit mask used in the MVPA analysis took into account also voxels in frontal and parietal cortices, activated by contrasting sound to baseline in the voice localizer task.

Even if an uncorrected threshold was used to build this mask, IFG and PrcG previously showed sensitivity to voices at a stricter threshold during a block-design voice localizer paradigm in a larger cohort of subjects (N = 92), and they have been referred to as “frontal voice areas” (FVAs)^40^. In this study we showed that a portion of the left IFG which was less than 8 mm apart from the middle left FVA, significantly classified speaker identity. Left IFG seems also to be implicated in sound learning^41^, tonal rehearsal^42^ and in processing phonetic structure of languages^43,44^. Hence, significant speaker identity classification accuracy could mirror phonological and mnemonic rehearsal of voices (delivered as verbal stimuli). According to the dual-stream model for auditory language processing, left IFG triangularis belongs indeed to the dorsal stream, connecting STC to prefrontal regions and sustaining articulatory-based speech coding rather than sound-to-meaning coding^45,46^.

Motor and somatosensory regions in the pre and post-central gyrus sustain speech production and vocal imitation^47,48^ but they have also been linked to neural sensitivity to voices^40^ and, more generally, to speech perception^30,49–52^. In our study, the cluster showing significant speaker classification accuracy at the border between pre and post central gyrus was located approximately where the posterior frontal voice area was observed in Aglieri et al.^40^ (Euclidean distance < 8 mm) and about 10 mm from where orofacial movements sustaining speech are encoded^53^, and could for instance mirror mental rehearsal of articulatory features associated to the words being heard. However, since subjects’ choice was performed through button press, it cannot be excluded that significant classification accuracy in this region reflects, to some extent, one-to-one mapping between speakers’ voice and finger tapping. Indeed, the present study did not aim to disentangle motor from auditory components of the speaker recognition task by separating in time auditory stimulus from motor response, or by using a controlled task requiring similar finger responses without speaker recognition. So the cortical regions highlighted by our analyses outside of auditory cortex probably do not represent a purely auditory, abstract representation of speaker identity but more likely a mixture of the auditory and motor components of the task to different unknown degrees.

The involvement of bilateral SMG in speaker classification seems to be in line with the observation of impaired voice identity recognition associated to lesions to right inferior parietal lobule/SMG^54^. Our finding of above-chance speaker classification accuracy in right SMG seems hence to confirm the importance of this region in discriminating between different voices. According to the current results, speaker classification accuracy was significantly above-chance also in left SMG/AG, a region involved in memorizing and retaining pitch^55–58^. Hence, it could be hypothesized that during the current task, left SMG/AG carried out speaker classification based on acoustical cues such as F0. However, further studies should clarify which acoustical cue drives such finding.

### Behavioural and neural differences in speaker recognition process

The fMRI studies that previously investigated the relationship between behavioural and neural inter-individual variability in voice recognition abilities employed different experimental paradigms, yielding contrasting results^8–10,59^. Watson et al.^59^ found for instance that unfamiliar voice recognition abilities correlated with sensitivity to undifferentiated sounds in right STG/STS but not with specific sensitivity to voices (as compared to non-vocal sounds). Andics et al.^8^ observed that BOLD activity related to identity processing during an fMRI task positively correlated with identification performance for previously learned voices in different regions: bilateral STS, left amygdala and right temporal pole. In Schelinski et al.^9^, the ability of healthy participants at recognizing speakers’ voices learned through the experiment correlated with BOLD signal in right STG during performance of the task. Bonte et al.^10^ found that classifier’s accuracy in discriminating between three speakers within left mid/posterior STS/STG increased with scores obtained at speaker identification. Our results are in line with this last study: higher voice identification abilities were associated with higher fMRI-based classification accuracy in left temporal pole. According to recent models of voice recognition^60^, the anterior temporal pole is part of the extended system for voice processing where information about known individuals, gathered through different perceptual modalities, could be contained. Even if the voices presented in the current study were not personally familiar to the subjects nor they belonged to celebrities, they were learned through different sessions, which probably allowed building an internal representation of the three speakers.

Importantly, fMRI-based speaker classification accuracy in left IFG also correlated with behavioural scores, explaining nearly half the variance (R^2^ = 0.45) in identification scores across participants. The highest classification accuracy score was observed in left IFG opercularis, identified as the middle frontal voice area in Aglieri et al.^40^. That region was actually the only one in which the correlation between the behavioural and fMRI-based confusion matrices approached significance, suggesting it hosts a representation of speaker identity closer to behavioural outcome than in temporal regions. This region, together with IFG triangularis, which showed significant fMRI-based classification accuracy, has a well-known role in speech processing^61,62^. IFG seems to have a role in higher-level identity processing: it is involved in face processing^63^ but also in cross-modal identity processing^64^. As put forward by Maguinness et al.^65^, the IFG, together with anterior temporal pole, could constitute the extended neural network of voice identity recognition; hence, the observation of a significant correlation between fMRI-based classification accuracy in voxels of this region and speaker identification scores supports the idea that inter-individual differences in voice recognition abilities could become evident in higher-level regions of the voice processing system, that possibly exert a top-down influence on auditory regions in the temporal cortex.

For the first time, we demonstrate that speaker classification accuracy within frontal regions can be related to behavioural differences in an online speaker identification task, at least when verbal stimuli are used. We recently reported correlations between behaviour and functional connectivity during a passive voice perception task, mostly between right frontal and temporal regions^40^. On the contrary, all correlations here observed were left-lateralized, as in Bonte et al.^10^. Even if the left hemisphere has been more often related to speech-related cues processing and right one to vocal processing^66–68^, individual differences in speaker identification are tightly related to the sensitivity to certain phonetic aspects of voices, such as formants, phonetic units dependent on speakers’ vocal tracts. For instance, Von Kriegstein et al.^69^ observed that activation of posterior portion of the left STS/STG was associated to both speech comprehension and changes in vocal tract parameters. To further support the importance of speech-related cues in discriminating the different speakers in the current study, it is worth reporting that first formant (F1) seems to highly contribute in discriminating female voices, in addition to fundamental frequency (F0)^12^. Furthermore, as reported by some subjects in the debriefing questionnaire, the way of speaking of the three speakers (e.g. rapidity, accent, linguistic prosody) as well as the manner of articulation, influenced their response. Hence, individual differences observed at this speaker identification task could depend on sensitivity to certain linguistic cues that are ecologically relevant for “telling speakers together and apart”^70^, in particular when F0 is particularly similar such as in the case of Betty’s and Chloe’s voices (see Supplementary Information).

However, even if we missed to observe a brain-behaviour correlation in right temporal regions, where pitch encoding is considered to take place^68^, it can be hypothesized that F0 was also an important acoustical cue for speakers’ recognition as indirectly demonstrated by the fact that Anne’s voice, whose F0 was the most different from the Betty’s Chloe’s, was the easiest one to recognize—and that subjects judged as more difficult discriminating Betty’s and Chloe’s voices (see Supplementary Information). Further studies could clarify which acoustical cues drive most individual differences in speaker recognition abilities and their neural correlates.

## Supplementary Information

Supplementary Information.
