# Supplementary material for: FMRI-based identity classification accuracy in left temporal and frontal regions predicts speaker recognition performance
Source: Sci Rep. 2021 Jan 12;11:489. doi: 10.1038/s41598-020-79922-7 (PMC7803954; doi:10.1038/s41598-020-79922-7)
Supplement: Supplementary file 1 — Supplementary Information. [file 41598_2020_79922_MOESM1_ESM.docx]

**FMRI-based identity classification accuracy in left temporal and frontal regions predicts speaker recognition performance**

Virginia Aglieri ^1^, Bastien Cagna ^1^, Lionel Velly ^1, 2^, Sylvain Takerkart ^1^, Pascal Belin ^1,3^

^1^ Institut de Neurosciences de la Timone, UMR 7289, CNRS and Aix-Marseille Université, 13005 Marseille, France

^2^ Department of Anesthesiology and Intensive Care, CHU Timone, Assistance Publique Hôpitaux de Marseille, Aix Marseille Université, 13005, Marseille, France

^3^ Department of Psychology, Université de Montréal, Montreal, QC H2V 2S9, Canada

**Supplementary Methods**

*Statistical analysis*

The total percentage of correct responses (PC) as well as speaker-specific PC scores were computed for each laboratory session (feedback 1, feedback 2, no feedback), for the pre-fMRI session and for each fMRI run. Missing responses in the identification task were considered as wrong responses. Chance level was always 33 % since there were three choices. Statistical analyses were performed in SPSS (IBM Corp. Released 2013. IBM SPSS Statistics for Windows, Version 22.0. Armonk, NY: IBM Corp) and Matlab R2014B (The MathWorks, Inc., Natick, MA, USA).

**Supplementary Results**

*Behaviour*

The distribution of PC scores obtained by the laboratory sample in the testing phase (M + SD = 69.6 + 15.3), on which subjects were selected for the fMRI session, is shown in Supplementary Figure 1A. Only one subject performed below chance level in the laboratory testing phase without any apparent cause (e.g. distraction); his score was included anyway in the analysis. Effects of session (n=3 - the two learning phases and the testing phase with no feedback) and speaker (n=3) as well as their interaction on identification scores were assessed through a repeated measure ANOVA with two 3-level within-subjects factors. In the laboratory session, there was a significant main effect of session on PC scores (*F* (2,170) = 16.23, *p* = 3.54e-7, η^2^ = 0.16). A main effect of speaker on PC scores was also present (*F* (1.91, 162.68) = 47.27, *p* < 4.75e-17, η^2^ = 0.36). Speaker-specific PC scores remained stable across the three sessions, as revealed by a non-significant interaction between the two within – subjects factors (*F* (4, 340) = 0.32, *p* = 0.86, η^2^ = 0.0004). A post-hoc Tukey test revealed that subjects’ identification scores were significantly worse in the first learning phase (feedback 1) than in both the second one (feedback 2; *p* = 2.52e-06) and in the testing phase (*p* = 6.46e-08), while from the second learning phase the performance was stable (*p* = 0.28). Post-hoc Tukey test yielded significant results for all comparisons between speaker-specific PC scores (all *p* < 0.001): Chloe’s voice was associated with worse recognition rate while Anne’s voice was the easiest one to recognize. This behavioural pattern characterized the 34 % of the subjects. Interestingly, participants’ subjective report assessed by the questionnaire delivered after the MRI session confirmed that most of them (48%) considered Chloe’s voice the most difficult to recognize. Most of the participants (67 %) also reported that Chloe’s and Betty’s voices were the most similar (see Suppl. Fig. 2).

Identification scores obtained in the fMRI session (N =40) are reported in Suppl. Table 1 and their distribution is shown in Suppl. Figure 1B. A repeated measure ANOVA was used to check for significant effects of speaker (3-levels within-subjects factor) and of run (4-levels within-subjects factor) on PC scores, reaction times and percentage of missing responses, and for a possible interaction between the two within-subjects factors. There was a main effect of speaker on both PC scores (*F* (2, 78) = 6.01, *p* = 0.003, η^2^= 0.13) and reaction times (*F* (1.61, 62.81) = 6.23, *p* = 0.006, η^2^= 0.14) while percentage of missing responses for different speakers were not significantly different (*F* (2,78) = 1.05, *p* = 0.35, η^2^= 0.03). There was no main effect of run on PC scores (*F* (3, 117) = 1.60, *p* = 0.19, η^2^ = 0.04), nor on reaction times (*F* (2.60, 101.44) = 1. 20, *p* = 0.29, η^2^ = 0.03), nor on percentage of missing responses (*F* (3, 117) = 1.22, *p* = 0.30, η^2^= 0.03), indicating that subjects were stable in their performance across the different runs. The interaction between run and speaker effects was not significant: the recognition rate specific for each speaker remained constant across runs (*F* (5.48, 213.73) = 1.14, *p* = 0.34, η^2 =^ 0.03), as well as the reaction times (*F* (6, 234) = 1.33, *p* = 0.24, η^2 =^ 0.03) and the percentage of missing responses (*F* (6, 234) = 0.91, *p* = 0.50, η^2 =^ 0.02). A post-hoc Tukey test revealed no significant differences between PC for Anne’s and Betty’s voices (p = 0.81), while Chloe’s voice was recognized significantly less accurately than both Anne’s (p = 0.004) and Betty’s voice (p = 0.02). As for reaction times, participants were significantly slower in recognizing Betty as compared to Anne (*p* = 0.006), while the other comparisons were not significant.

A repeated measure ANOVA was then used to assess the effect of the within subjects factor “experimental session” (laboratory, training and fMRI) and gender (between-subject factor), as well as their interaction, on overall performance (PC score obtained in the testing phase in the laboratory, mean of the two results obtained in the pre-fMRI training session and mean of the four fMRI runs). The comparison between the PC scores obtained in the three experimental session (no feedback session in the laboratory, pre-fMRI training and fMRI) revealed a main effect of session (*F* (2, 76) = 11.9, *p* = 0.00003, η^2^ = 0.24) while there was no significant interaction between session and gender (*F* (2, 76) = 0.33, *p* = 0.72, η^2^ = 0.009). Importantly, subjects’ performance was not significantly different between pre-fMRI training and fMRI session, as demonstrated by a post-hoc Tukey test (*p* = 0.76): subjects had apparently reached a stable performance level when entering the scanner. The results obtained in the fMRI session were instead significantly worse (*p* = 0.001) than those obtained in the first laboratory session in the no feedback condition, based on which subjects were selected. This is likely explained by the adverse acoustical environment of the fMRI scanner, despite precautions to insulate subjects from the fMRI scanning noise.

A linear regression model was used to assess if subjects’ performance in the laboratory predicted MRI behavioural scores and if interval of days separating laboratory and fMRI sessions explained performance in the scanner. This analysis established that the performance obtained in the laboratory session significantly predicted scores in the fMRI session (*F* (1, 37) = 42.03, *p* < 0.001, η^2^ = 0.53), accounting for 52 % of the explained variability in PC scores in the scanner (Suppl. Fig 1C). A linear regression established that the number of days separating laboratory and fMRI session did not significantly predict identification scores in the fMRI session (*F* (1, 37) = 2.10, *p* = 0.2, η^2^ = 0.05), accounting for only 0.1 % of the explained variability in PC scores.

Finally, the number of words recalled pronounced by the speakers in the fMRI session was used to test a relationship with the scores obtained at speaker identification in the scanner task, in order to test if memory for words influenced task performance. The number of words recalled after the fMRI session did not significantly predict PC scores in the fMRI session (*F* (1, 37) = 0.54, *p* = 0.5, η^2^ = 0.01), accounting for only 0.01 % of the explained variability.

The average responses obtained at the MRI questionnaire assessing behavioural aspects of the task (e.g. number of words recalled, subjective impressions on the tasks) are reported in Suppl. Table 2 and Suppl. Figure 2.


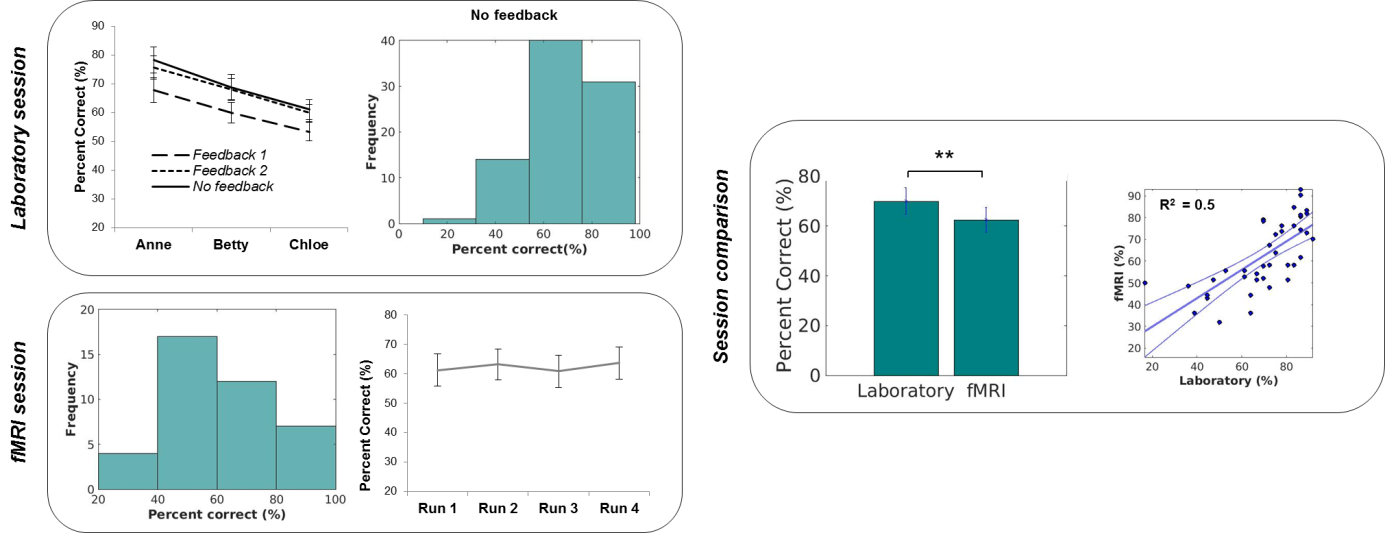


Supplementary Figure 1. Behavioural results. A. Laboratory session: performance obtained in the two learning phases (Feedback 1 & Feedback 2) and the testing phase (No feedback) for each speaker (left), and distribution obtained in the testing phase (right) on which subject selection for the fMRI session was performed. B. fMRI session: distribution of the scores obtained in the scanner (N = 40; left) and mean performance across the four runs (right). C. Session comparison: histogram (left) showing mean performance obtained in the lab (no feedback condition) and fMRI session (error bars = 95 % confidence interval) and correlation between the scores obtained in the two sessions (right). R^2^ = proportion of variance explained.

|  | **PC identification (lab)** | **PC identification (scanner)** | **Reaction time (sec) (scanner)** |
| --- | --- | --- | --- |
| **Anne** | 75.0 + 23.6 | 66.3 + 20.0 | 1.2 + 0.5 |
| **Betty** | 68.1 + 21.3 | 63.7 + 16.6 | 1.3 + 0.5 |
| **Chloe** | 62.7 + 16.3 | 56.5 + 16.3 | 1.3 + 0.4 |
| **Total** | 69.9 + 17.4 | 62.4 + 16.1 | 1.3 + 0.5 |

Supplementary Table 1.Behavioural results of main interest. Mean percent correct scores (PC) with relative standard deviation obtained by the laboratory sample in the testing phase where no feedback was delivered after response (1^st^ column) and in the fMRI session by the sample of 40 subjects, averaged over the four runs (2^nd^ column). 3^rd^ column: mean reaction times with relative standard deviations obtained in the fMRI session (averaged over the four runs).

|  | Number of words recalled | Do you think that the accent influenced your words comprehension?  [1 = not at all; 5 = extremely] | Did you think you correctly learned the name-voice association after the first two training sessions?  [1 = not at all; 5 = extremely] | Do you think you forgot the voices between laboratory and MRI session?  [1 = not at all; 5 = extremely] |
| --- | --- | --- | --- | --- |
| Mean + SD | 4.07 + 1.86 | 2.25 + 1.28 | 3.25 + 0.67 | 2.85 + 1.11 |

Supplementary Table 2. Results obtained at the post-MRI questionnaire assessing different aspects of the experiment.


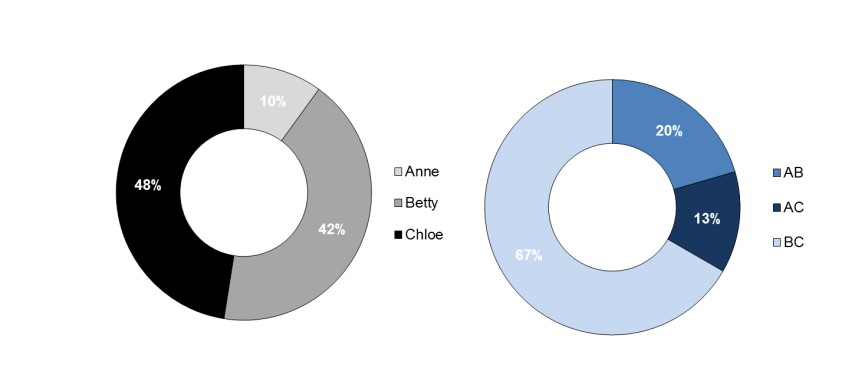


Supplementary Figure 2. Pie chart showing participants’ answers to the debriefing question “Whose voice was the most difficult to recognize for you?” (left) and “Whose voices were the most similar in your opinion?” (right; AB = Anne-Betty; AC = Anne-Chloe; BC = Betty-Chloe).
